# TRIP13 regulates progression of gastric cancer through stabilising the expression of DDX21

**DOI:** 10.1038/s41419-024-07012-x

**Published:** 2024-08-26

**Authors:** Guanghui Zhang, Rui Yang, Baiyan Wang, Qiujin Yan, Peiyuan zhao, Jiaming Zhang, Weiyu Su, Lianhe Yang, Hongjuan Cui

**Affiliations:** 1 Medical College, Henan University of Chinese Medicine, Zhengzhou, China; 2 Biomedical Laboratory, School of Medicine, Liaocheng University, Liaocheng, China; 3 Cancer Center, Medical Research Institute, Southwest University, Chongqing, China

**Keywords:** Oncogenes, Tumour biomarkers

## Abstract

GC (Gastric cancer) is one of the most common malignant tumours, with over 95% of gastric cancer patients being adenocarcinoma and most gastric cancer patients having no apparent symptoms in the early stages. Finding biomarkers for early screening of gastric cancer and exploring new targets for gastric cancer treatment are urgent problems to be solved in the treatment of gastric cancer, with significant clinical outcomes for the survival rate of gastric cancer patients. The AAA+ family ATPase thyroid hormone receptor-interacting protein 13 (TRIP13) has been reported to play an essential role in developing various tumours. However, the biological function and molecular mechanism of TRIP13 in gastric cancer remain unclear. This study confirms that TRIP13 is highly expressed in gastric cancer tissue samples and that TRIP13 participates in the proliferation, migration, invasion in vitro, and tumourigenesis and metastasis in vivo of gastric cancer cells. Mechanistically, this study confirms that TRIP13 directly interacts with DDX21 and stabilises its expression by restraining its ubiquitination degradation, thereby promoting gastric cancer progression. Additionally, histone deacetylase 1 (HDAC1) is an upstream factor of TRIP13, which could target the TRIP13 promoter region to promote the proliferation, migration, and invasion of gastric cancer cells. These results indicate that TRIP13 serve is a promising biomarker for the treating of gastric cancer patients, and the HDAC1-TRIP13/DDX21 axis might provide a solid theoretical basis for clinical treatment of gastric cancer patients.

## Background

Gastric cancer is the fifth most common cancer worldwide and the third leading cause of cancer death [1]. Gastric cancer is an aggressive and heterogeneous malignant tumor, with a median overall survival (OS) of 16 months for patients [1, 2]. The identified risk factors for gastric cancer include diet and lifestyle factors, such as Helicobacter pylori infection, smoking and obesity, as well as genetic mutations and instability. Laparoscopic-assisted distal gastrectomy and traditional open distal gastrectomy have been recommended as standard treatment options for early gastric cancer. Despite significant improvements in diagnosis and treatment strategies, as well as patient survival rates, patients with lower malignancy in the early stage of gastric cancer are usually asymptomatic [3], and many patients are observed to be in the late stage during diagnosis, leading to poor prognosis and high recurrence rates. Therefore, better elucidating the pathogenesis of gastric cancer, searching for biological biomarkers for early screening of gastric cancer, and exploring new targets for gastric cancer treatment are urgent issues that need to be addressed in the current treatment of gastric cancer, with important clinical significance for improving the survival rate of gastric cancer patients.

The thyroid hormone receptor interaction factor 13 (TRIP13) gene encodes a protein that interacts with thyroid hormone receptors, also known as hormone-dependent transcription factors. The gene product undergoes specific interactions with ligand domains [4]. TRIP13 has been shown to regulate the embryo sac, monitoring the attachment of kinetochore microtubules during mitosis and meiosis [5]. TRIP13 is a member of the ATPase family associated with various cellular active proteins and is highly conserved in many species. Previous studies have shown that TRIP13 is a new spindle assembly checkpoint (SAC) pathway component, crucial for the precise distribution of duplicated chromosomes [6]. Studies indicate that depletion of TRIP13 induces insulin receptor/Akt pathway-dependent adipogenesis. A decrease in TRIP13 levels leads to lipid droplet accumulation and functional aMTOCs interference with spindle polarity during mitosis, leading to tumour cell death [7]. Researchers have also investigated the role of Mad2 and TRIP13 in inhibiting Aurora kinase-induced HPV-positive retinoblastoma cell apoptosis. Studies have demonstrated that the combination of TRIP13 deficiency and Aurora kinase inhibition resulted in significantly more cell apoptosis than selective single-pathway inhibition [8]. The discovery might contribute to the development of new therapies for the treatment of retinoblast Rb deficient cancers. In addition, TRIP13 plays a crucial role in meiotic recombination and DNA repair in plants, yeast, worms, and mice [9, 10]. More and more studies have shown that the abnormal activation of TRIP13 promotes non-homologous terminal connections [11], and its overexpression leads to cell transformation and resistance to chemotherapy drugs. These studies indicate that TRIP13 might be related to tumour progression and speculate that it is a promising biomarker and potential therapeutic target for cancer diagnosis and treatment. Previous studies have shown that TRIP13 plays an oncogene role in various tumours and is involved in tumour cell proliferation, apoptosis, migration, and invasion, including colorectal cancer, head and neck cancer, glioblastoma, cervical cancer, multiple myeloma, hepatocellular carcinoma, and cholangiocarcinoma [8, 12–17]. In addition, TRIP13 was found to be involved in the activation of AKT/mTOR, Wnt/β-catenin and TGF-β1/SMAD3 signalling pathways to promote the proliferation and metastasis of tumours [16, 18, 19]. The small molecule inhibitor DCZ0415 targeting TRIP13 has been reported to restrain the multiplication ability of multiple myeloma cells, colorectal cancer cells, pancreatic ductal adenocarcinoma cells, and lung adenocarcinoma cells. Existing studies have revealed that TRIP13 is highly expressed in gastric cancer and participates in the proliferation and apoptosis of gastric cancer cells [20, 21]. However, the molecular mechanisms by which TRIP13 participates in gastric cancer proliferation, tumourigenesis and metastasis remain unclear.

RNA binding proteins (RBPs) are a type of characteristic protein used for phase separation. RBP simultaneously possesses RNA recognition motifs (RRM) and IDR interactions with RNA, which promote and stabilise their phase separation condensates [22]. The phase separation of RBPs is often observed in RNA granules, RNA spots, and nucleoli, which are deeply involved in RNA transcription, splicing, and modification [23, 24]. As the most prominent RNA helicase family, members of the DEAD/H-BOX family are representative RBPs. There are 59 types of DEAD/H-box helicases [25], including 44 DEAD box (DDX) helicases and 15 DEAH box helicases, which are involved in most RNA physiological processes, such as RNA transcription, editing, splicing, and transport [22, 26]. DDX21, as an RNA helicase, is primarily involved in regulating of ribosomal RNA synthesis and ribosomal biogenesis. Reports have shown that the function of DDX21 is crucial for VEGFC-FIT4-driven endothelial cell proliferation. Without DDX21, endothelial cells exhibit reduced ribosomal biogenesis, upregulation of P53 and P21, and cell cycle arrest that blocks lymphatic vessel formation [27]. Such correlations demonstrate that DDX21 plays a role as a cancer-promoting factor. Other studies have also confirmed that the RNA binding protein DDX21 is highly expressed in colorectal cancer, and promotes the migration and invasion ability of colorectal cancer cells through high binding to the MCM5 locus and interaction with the cell division cycle-related protein CDC5L, respectively [22, 28]. Recent studies have confirmed that DDX21 is highly expressed in gastric cancer and is ubiquitinated through deubiquitinase USP10 [29]. Regarding the oncogene role of DDX21 in gastrointestinal tumours such as colorectal cancer and gastric cancer, we found that TRIP13 and DDX21 bind to each other through mass spectrometry analysis and related experiments, and TRIP13 could promote the development of gastric cancer by stabilising the expression of DDX21. Such results provide new perspectives and targets for the clinical treatment of gastric cancer patients.

The current study demonstrates that TRIP13 is abnormally activated in gastric cancer, and the immunohistochemistry assay reveals that as the grade of gastric cancer increases, the expression of TRIP13 also increases. Depletion of TRIP13 in gastric cancer cells reduces proliferation, tumour growth, and tumour metastasis, inducing a cell cycle arrest, which is rescued by the reconstituted expression of TRIP13. Mechanically, the current study confirms that TRIP13 interacts with DDX21, and mediates the inhibition of ubiquitination degradation of the latter, thereby facilitating the progression of gastric cancer. Furthermore, histone deacetylase 1 (HDAC1) is found to act as an upstream factor of TRIP13, promoting its transcriptional activation. Overall, this study reveals the reasons for the abnormal activation of TRIP13 in gastric cancer, inferring that TRIP13 might become a biomarker for early diagnosis of gastric cancer. HDAC1-TRIP13/DDX21 axis provides a promising theoretical basis for treating gastric cancer patients.

## Materials and methods

### Cell lines and cell culture

All human gastric cancer cell lines (MKN45, HGC27), and human embryonic kidney (HEK) 293T cells were obtained from the American Type Culture Collection. The above cells were cultured in 1640 and DMEM medium with 10% foetal bovine serum (FBS). All cell lines were authenticated by short-tandem repeat analysis. These cell lines were mycoplasma-free (checked using a Mycoplasma Stain Assay Kit named C0296, Beyotime Biotechnology, Shanghai, China) and cultured in a humidified environment supplemented with 5% CO_2_ at 37 °C.

### Reagents, antibodies, and clinical tissue samples

MG132 and CHX were obtained from Sigma (Shanghai, China). A rabbit-enhanced polymer detection system (#PV-9001) for immunohistochemistry (IHC) was purchased from ZSGB-Bio (Beijing, China). The antibodies used include the following: 19602-1-AP (anti-TRIP13), 10528-1-AP (anti-DDX21), 66925-1-Ig (anti-DDX21), 10122-1-AP (anti-CDK2), 11224-1-AP (anti-α-Tubulin), 11554-1-AP (anti-CCNE1), 51064-2-AP (anti-HA-Tag), 20543-1-AP (anti-Flag-Tag), 27309-1-AP (anti-Ki67), 20874-1-AP (anti-E-cadherin), 22018-1-AP (anti-N-cadherin) and 10197-1-AP (anti-HDAC1) were purchased from Proteintech (Wuhan, China), ab216347 (anti-Snail) was obtained from Abcam (Shanghai, China). Clinical gastric cancer tissue samples were purchased from Zhongke Guanghua (Xi’an) Intelligent Biotechnology Co., Ltd.

### Immunohistochemistry staining

Paraffin-embedded tumours were cut into slices with a thickness of 6 mm, and then the paraffin sections were dewaxed and hydrated. Then, paraffin slices were put into citrate buffer (pH 6.0) and heated in a microwave oven to 95 °C for 20 min to facilitate antigen retrieval. Then, endogenous peroxidase activity was quenched, followed by blocking with normal goat serum. Then, the TRIP13, Ki67 and DDX21 antibodies were diluted with PBS (1:150), and the antibodies were added to the paraffin sections and incubated overnight at 4 °C. Then, a horseradish peroxidase-linked secondary antibody was added and incubated with the sections, followed by a DBA reagent. Finally, the paraffin sections were stained with hematoxylin, dehydrated, and sealed with a cover glass. Observe the experimental results under a microscope.

### Plasmids, lentivirus packaging, and infection experiments

Small-hairpin shRNAs for TRIP13, HDAC1 and DDX21 and a negative control shRNA (shGFP) were obtained from Gene Pharma Co. Ltd. (Shanghai, China) and were inserted into the pLKO.1 vector (Addgene_13425). Flag-TRIP13, MYC-DDX21 and HA-UB were cloned into the pCDH-CMV-MCS-EF1-GFP-Puro vector (Addgene_60358), which was purchased from Youbao Company (Changsha, China). For lentivirus packaging and infection assays, the target plasmid (500 ng) and packaging plasmid (PLP1-Addgene_209988, PLP2-Addgene_209989, and VSVG-Addgene_8454)/500 ng were co-transfected into 293T cells in one six-well plate by using the transfection reagent Lipofectamine 2000 (Invitrogen, Carlsbad, CA, USA). Lentiviruses were collected 48 h later and used to infect gastric cancer cells twice, 12 h per infection. The infected cells were screened by treatment for 48 h with puromycin, and the surviving cells were frozen and stored in liquid nitrogen for subsequent assays. The multiplicity of infection (MOI) value of lentivirus was 36. The laboratory of the Research Center of the School of Medicine at Henan University of Chinese Medicine meets the biosafety standards for lentivirus preparation. After conducting experiments related to lentivirus packaging, and infection experiments, the experimental supplies were subjected to high-pressure sterilization treatment. During the packaging process of lentiviruses, replication-competent lentivirus (RCL) might be generated due to homologous or non-homologous recombination mechanisms. For safety, effectiveness, and controllable quality considerations, replication-competent lentivirus (RCL) detection kit (41311ES50) was obtained from YEASEN Biotechnology Co., Ltd (Shanghai, China) for measuring the potential risks of replication-competent lentivirus that might occur in cells related to lentiviral packaging assay. The detection results indicate that there is no potential risk of replication-competent lentivirus. The shRNA primer pairs are shown in Supplementary Table 2.

### Silencing and rescue experiments

Small-hairpin shRNAs for TRIP13 were obtained from Gene Pharma Co. Ltd (Shanghai, China) and were inserted into the pLKO.1 vector (Addgene_13425). TRIP13 with Flag tag targeting shTRIP13 sequence synonymous mutations were cloned into the pCDH-CMV-MCS-EF1-GFP-Puro vector (Addgene_60358), which was purchased from Youbao Company (Changsha, China). As mentioned above, lentivirus packaging experiments should be conducted to collect shTRIP13 and Flag-TRIP13 lentiviruses. Then, gastric cancer cells were infected with shTRIP13 lentivirus and screened through puromycin. The surviving cells were further infected and screened with Flag-TRIP13 lentivirus for subsequent Edu assay, plate clone formation assay, and transwell assay.

### Animal models and tumour xenograft

Four-week-old female nude mice were purchased from Huafukang Biotechnology Co., Ltd. Mice were randomly assigned to receive subcutaneous injection and divided into four groups (6 mice/group) and two groups (5 mice/group). In short, stably transfected gastric cancer cells MKN45 (shGFP, shTRIP13, shTRIP13/TRIP13, shTRIP13/DDX21, and shDDX21) (1 × 10^6^ cells) and resuspend them on 100 μl Inject PBS into the subcutaneous tissue of mice. Before subcutaneous injection, anesthetise the mice with an isoflurane nasal anesthesia system to alleviate pain. Before collecting tumours, anesthetise mice with an isoflurane nasal anesthesia system and euthanise them through cervical dislocation. Then, the mice corpses are collected, stored at −20 °C, and send them to the limited company Leibit Biotechnology Co., Ltd. for incineration. The tumor volume is calculated using the following formula: V= (length × width^2^)/2. The tumors were collected and photographed for subsequent immunohistochemical experiments. As mentioned above, four-week-old female mice were randomly divided into three groups (4 mice/group). Stable transfected gastric cancer cells MKN45 (shGFP, shTRIP13, shTRIP13/TRIP13) (4 × 10^6^ cells) were resuspended in 80ul PBS and injected into the mice via the tail vein. Six weeks later, anesthetise mice with an isoflurane nasal anesthesia system and euthanise them through cervical dislocation. Collect mouse lung tissue for subsequent H&E staining assay. Finally, macroscopic metastasis was quantified by counting the lesions in all lung lobes of each mouse.

The Animal Protection and Ethics Committee of Henan University of Chinese Medicine approved all animal treatments with approval number IACUC-S202403084. All experiments were conducted per the Ministry of Science and Technology of China’s Animal Health and Use Guidelines (2006).

### Western blot assay

For the western blot assay, cells were retrieved and lysed on ice by RIPA lysis buffer (Beyotime_P0013J). The supernatant was collected after centrifuging at 12,000 rpm at 4 °C for 10 min. The protein was then denatured in a 100 °C water bath, followed by SDS-PAGE and transmembrane (Bio-Rad, USA: 68505100), sealed with 5% skimmed milk powder at room temperature for 2 h. The secondary antibody (Proteintech_SA00001-2, China) was incubated after the primary antibody’s incubation, with the membrane exposed in the gel imaging system (Qinxiang, China).

### Immunoprecipitation (IP) assay

The IP experiment was conducted as previously described [30]. Briefly, cells are collected and lysed through an IP lysis buffer (Beyomime_P0013J). The supernatant was collected following centrifugation, at 12,000 rpm 4 °C for 10 min. The supernatant and Protein A/G magnetic beads (Beyomime_P2012) with anti-TRIP13 (Proteintech_19602-1-AP) and anti-DDX21 (Proteintech_10528-1-AP) respectively, are placed on a shaking bed at 4 °C overnight. Simultaneously, IgG (Beyomime_A7016) was used as the negative control group for the same procedure. After overnight incubation at 4 °C, the Protein A/G magnetic beads were washed with phosphate buffer to remove non-specific protein binding. Then, denature at 100 °C, followed by SDS-PAGE and transmembrane (Bio-Rad, USA: 68505100). Incubate the primary antibody overnight, and then incubate the secondary antibody (Thermo Fisher_21230) for western blot analysis.

### Ubiquitination and turnover assay

The constructed plasmids shGFP, shTRIP13, Flag-TRIP13 and HA-UB plasmid transfection into 293T cells. Forty-eight hours after transfection, 50 µg/ml proteasome inhibitor MG132 was added to the cells and incubated for 8 h. The cells were collected and lysed using an IP lysis buffer. The cell lysate was incubated at 4 °C with antibody-conjugated beads. The beads were washed, denatured at 100 °C, and the proteins were detected using Western blotting. For the turnover assay, infected cells were subjected to 48 h and treated with CHX at a concentration of 50 µg/ml cells. Finally, cells were collected, lysed, and analysed using western blot.

### Edu staining

According to the manufacturer’s recommendation, an Edu staining assay was performed to detect cell proliferation [31]. 2 × 10^4^ cells were placed in a 24-well plate and cultured overnight. The next day, the cells were first incubated with 10 mM Edu for 2 h, followed by incubation with 4% paraformaldehyde (PFA) for 15 min, incubation with 0.3% Triton X-100 for 10 min, and incubation with 5% bovine serum albumin (BSA) for 1 h. Before taking photos under a microscope for processing, the nuclei were stained with DAPI at room temperature for 30 min.

### Cell proliferation detection and plate colony formation experiment

To detect cell proliferation ability, 1 × 10^3^ stable transfected MKN45 and HGC27 cells were cultured in 96 and 6 well plates for 6 and 15 days, respectively. As mentioned previously [32], cell viability was detected using cell counting and MTT assays. After staining with crystal violet, the scanning plate clone formation assay was used, and the absorption rate of 560 was finally measured using absolute ethanol to measure nm for statistical analysis. All experiments were conducted independently three times.

### Quantitative reverse transcriptional PCR (qRT-PCR)

As mentioned previously [33], using TRIzol reagent (Invitrogen)™), Reverse transcribe 2 µg of RNA into cDNA. SYBR qPCR Super Mix Plus is used for qRT PCR (Novoprotein, China). The RT-PCR primer pairs are shown in Supplementary Table 1. Results were calculated using the ΔΔCt method with glyceraldehyde-3-phosphate dehydrogenase as the internal control [34].

### Luciferase reporter assay

The promoter region of TRIP13 is connected to the pGL3 vector and obtained from Changsha Youbao Biotechnology Co., Ltd. Then, by transfecting with Lipofectamine 2000, the pGL3 plasmid, pRL-TK internal control vector (Promega), and shHDAC1 vector were co-transfected into MKN45 and HGC27 cells. After 48 h, luciferase reporter gene testing was performed according to the manufacturer’s instructions (Promega). Each group has three repeated experiments.

### Proximity ligation assay (PLA) and immunofluorescence (IF) assay

Grown on a cover glass slide in a 24-well plate, 3 × 10^4^ MKN45 and HGC27 cells were transiently transfected, followed by fixation in 4% paraformaldehyde for 20 min and permeabilisation in 0.3% Triton X-100 at room temperature for 15 min. Then, after sealing in 5% goat serum at room temperature for 1 h, incubate the cover glass with primary antibody overnight at 4 °C. The subsequent experiments were conducted using the PLA assay kit [35]. Finally, the cells were visualised and photographed using a confocal fluorescence microscope.

### Migration and wound healing assays

Cell migration and invasion experiments were conducted using transwell chambers (pore size 8 µm, Corning, Beijing, China). For intrusion experiments, cover the membrane with Matrix (BD Biosciences). Add 1640 medium with 10% fetal bovine serum to the lower part of the chamber, and add cells from serum-free 1640 medium to the upper part of the chamber. After 25 and 29 h, stable transfected MKN45 and HGC27 cells were fixed with 4% paraformaldehyde for 15 min, and stained with crystal violet. Calculate the average number of cells from at least three randomly selected microscope images. For cell wound healing determination, culture cells in a six-well plate and make the wound using the tip of a 10 µl pipette. Finally, the healing process of cells is observed through a microscope.

### Patient database analysis

All data sets were obtained from GEPIA (http://gepia.cancer-pku.cn/index.html) databases, TIMER2 (http://timer.cistrome.org/), R2 databases (https://hgserver1.amc.nl/cgi-bin/r2/main.cgi), gastric cancer (Kaplan–Meier Plotter) databases and Protein Atlas database (http://www.proteinatlas.org/).

### Statistical analysis

Triplicates at least were designed and performed in each of the above experiments. Statistical parameters (sample size and significance analysis) are shown in the legend. Data were analysed and shown as mean ± SD. Statistics analyses were performed using GraphPad Prism 8.0. Two-tailed Student’s t-tests were performed for paired samples. *p < 0.05, **p < 0.01, and ***p < 0.001 were considered to indicate statistical significance.

## Results

### TRIP13 is highly expressed in gastric cancer and is associated with a poor prognosis

**Fig. 1:**
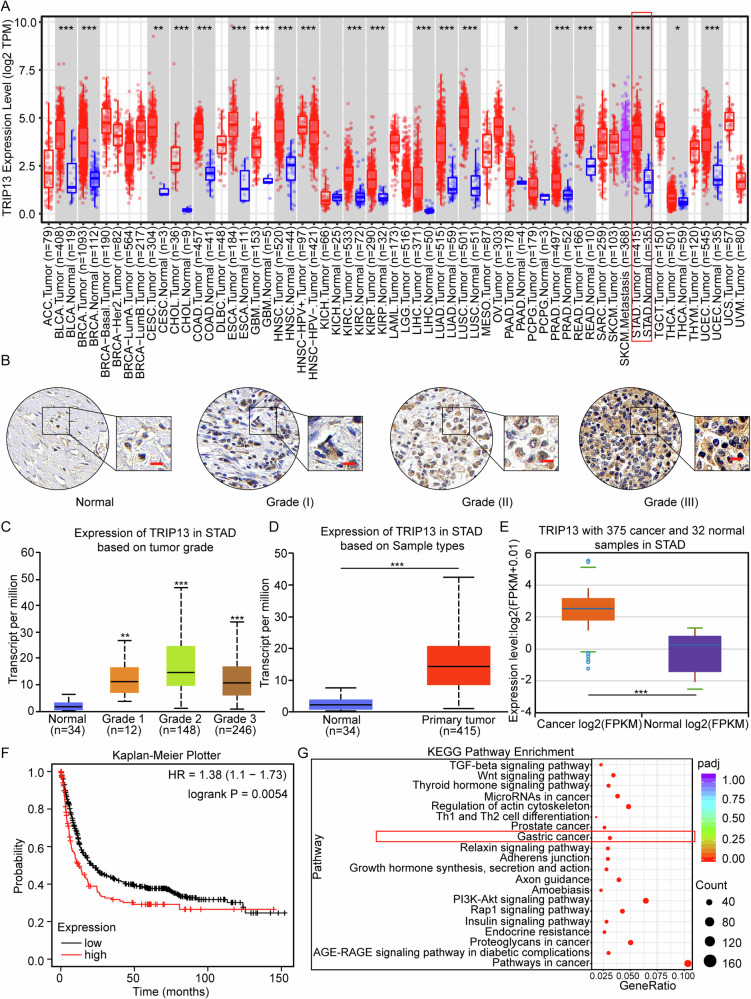
TRIP13 is highly expressed in gastric cancer and is associated with a poor prognosis. **A** Expression of TRIP13 in gastric cancer was determined through the TIMER2 (http://timer.cistrome.org/) database. **B** Immunohistochemical experiments were conducted to detect the expression of TRIP13 in normal gastric mucosal tissue and different gastric cancer tissue samples. **C** UALCAN database (https://ualcan.path.uab.edu/cgi-bin/ualcan-res.pl) was used to detect the expression of TRIP13 in normal gastric mucosal tissue and samples from patients with different levels of gastric cancer. **D**, **E** The GEPIA database (http://gepia.cancer-pku.cn/) and ENCORI database (https://rnasysu.com/encori/) were used to detect the expression of TRIP13 in normal gastric mucosal tissue and gastric cancer patient samples. **F** The Kaplan–Meier plotter database (https://kmplot.com/analysis/) was applied to detect prognostic significance of TRIP13. **G** Analysis of transcriptome database indicates that TRIP13 could be enriched in gastric cancer. All data were expressed as mean ± SD. The student’s *t*-test was performed to analyse the significance. *p < 0.05, **p < 0.01, ***p < 0.001.

The expression of TRIP13 was analysed through the TCGA data set, to determine the function of TRIP13 in gastric cancer. Database analysis demonstrates that TRIP13 is highly expressed in gastric cancer and various other tumours (Fig. 1A). Subsequently, the expression of TRIP13 was detected in normal gastric mucosal tissue and gastric cancer tissue samples of different grades through immunohistochemistry experiments. The results validate that TRIP13 was highly expressed in gastric cancer tissue samples, and the expression of TRIP13 increased with the increase of gastric cancer grade (Fig. 1B). Additionally, analysis of the UALCAN and ENCORI databases reveals that TRIP13 is highly expressed in gastric cancer (Fig. 1C–E). Next, the prognosis of TRIP13 in gastric cancer was analysed using the Kaplan–Meier plotter. The results indicate that high expression of TRIP13 results in poor prognoses for gastric cancer patients. The above results demonstrate the abnormal activation of TRIP13 with poor prognosis in patients (Fig. 1F). To further investigate the biological function of TRIP13 in gastric cancer, transcriptomic analysis is undertaken on gastric cancer cells with knockdown of TRIP13 expression. KEGG enrichment analysis in transcriptomics shows significant enrichment in the PI3K/AKT signaling pathway and gastric cancer after knocking down TRIP13 (Fig. 1G). In combination, these results indicate that TRIP13 is abnormally activated in gastric cancer and is significantly associated with poor prognosis in gastric cancer patients.

### TRIP13 is essential for cell proliferation in Gastric cancer

**Fig. 2:**
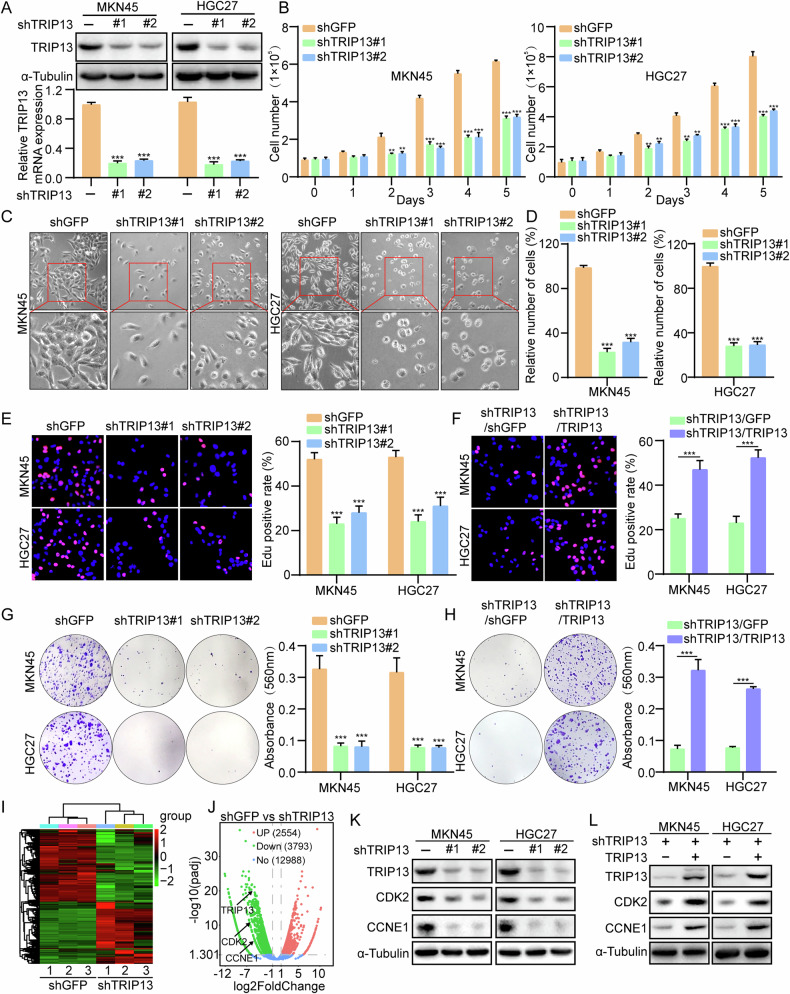
TRIP13 is essential for cell proliferation in Gastric cancer. **A** qRT-PCR assay and western blot assays were performed to detect TRIP13 expression in TRIP13-knocked-down MKN45 and HGC27 cells. **B** Growth experiment was used to detect the effect of knocking down TRIP13 on the proliferation ability of gastric cancer cells. **C**, **D** Morphology and numbers of cells were observed after knocking down TRIP13. **E** Cells positive for Edu staining were shown in knocking down TRIP13 MKN45 and HGC27 cells. **F** Cells positive for Edu staining after overexpression of TRIP13 in TRIP13-knockdown gastric cancer cells are shown. **G** Clone-forming ability was observed in knocking down TRIP13 gastric cancer cells. **H** Colony-formation was performed in TRIP13-overexpressing in TRIP13-knockdown gastric cancer cells. **I** The volcano plot analysis of transcriptomics revealed the number of upregulated and downregulated genes in MKN45 cells after knocking down TRIP13. **J** Western blot was performed to detect the protein expression of TRIP13, CDK2, and CCNE1 in TRIP13 knockdown cells. **K** Western blot was performed to detect the protein expression of TRIP13, CDK2, and CCNE1 in TRIP13-overexpressing in TRIP13-knockdown gastric cancer cells. All data were expressed as mean ± SD. The student’s *t*-test was performed to analyse the significance. *p < 0.05, **p < 0.01, ***p < 0.001.

A stable gastric cancer cell line knocking down TRIP13 using lentiviral interference technology (Fig. 2A) was developed to explore the effect of TRIP13 on the proliferation ability of gastric cancer cells. In gastric cancer cells knockdown TRIP13, knockdown of TRIP13 significantly restrains the growth of gastric cancer cells (Fig. 2B). Cells with TRIP13 knockdown exhibit marked morphological differences, and the number of cells is significantly decreased (Fig. 2C, D). The Edu assay and plate clone formation assay revealed that knocking down TRIP13 significantly repressed the proliferation of gastric cancer cells (Fig. 2E, G). To investigate whether the knockdown of TRIP13-induced inhibition of gastric cancer cell proliferation is not caused by off-target effects, rescue experiments involving Edu and plate cloning assays were undertaken. The results indicate that the expression of rescue TRIP13 significantly restores the proliferation inhibition of gastric cancer cells caused by knocking down TRIP13 (Fig. 2F, H). According to transcriptomic enrichment analysis, it was found that knocking down TRIP13 significantly enriched genes related to the cell cycle (Fig. 2I). Western blot experiments reveal significant downregulation of cell cycle-related proteins CDK2 and CCNE1 after knocking down TRIP13. After rescuing the expression of TRIP13, the expression of cell cycle-related proteins is significantly rescued (Fig. 2J, K), (Fig. S3A, B). Additionally, the small molecule inhibitor DCZ0415 targeting TRIP13 could repress the proliferation of gastric cancer cells through plate cloning and Edu assays (Fig. S1A, B). The above experimental results reveal that TRIP13 is necessary for the proliferation of gastric cancer cells and might play an oncogene role.

### TRIP13 facilitates gastric cancer cell migration and invasion in vitro and metastasis in vivo

**Fig. 3:**
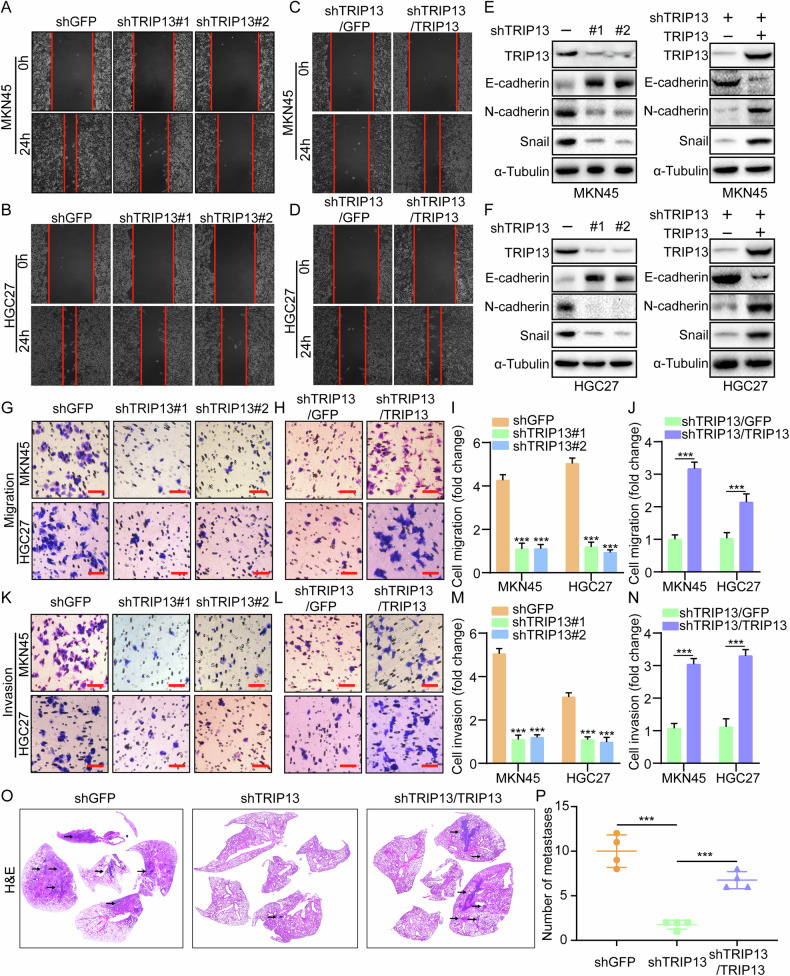
TRIP13 facilitates gastric cancer cell migration and invasion in vitro and metastasis in vivo. **A**, **B** Wound-healing assay was performed with TRIP13-knockdown MKN45 and HGC27 cells. **C**, **D** Wound-healing experiment was conducted in gastric cancer cells that rescued TRIP13 expression after knocking down TRIP13. **E**, **F** Western blot experiments were performed to detect the protein expression of TRIP13, E-cadherin, N-cadherin and Snail in gastric cancer cells that were knocked down and rescued after knocking down TRIP13. **G**–**N** Transwell assay was performed to detect the migration and invasion of gastric cancer cells that were knocked down and rescued after knocking down TRIP13. **O**, **P** The tail vein injection assay in mice was adopt to detect the effects of knocking down TRIP13 and rescuing TRIP13 expression after knocking down TRIP13 on the in vivo metastasis ability of gastric cancer cells in mice. All data were expressed as mean ± SD. The student’s *t*-test was performed to analyse the significance. *p < 0.05, **p < 0.01, ***p < 0.001.

High aggressive ability is one of the essential reasons for the difficulty in treating of gastric cancer patients. To explore the effect of TRIP13 on the migration and invasion in vitro and metastasis in vivo of gastric cancer cells, several cell scratch, transwell and mice tail vein injection assays were conducted. The cell scratch assay and transwell assay revealed that knocking down TRIP13 significantly reduced gastric cancer cell migration and invasion ability (Figs. 3A–D and S2A, B). Knocking down TRIP13 and rescuing its expression could restore the inhibition of gastric cancer cell migration and invasion caused by knocking down TRIP13 (Fig. 3G–N). Western blot assays demonstrated that migration and invasion-related proteins N-cadherin and Snail are down-regulated after TRIP13 knockdown, whereas the expression of E-cadherin is increased in TRIP13-knockdown cells (Fig. 3E, F). After rescuing the expression of TRIP13, migration invasion-related proteins exhibited significant recovery (Figs. 3E, F and S3A, B). Finally, a tail vein injection experiment in mice was undertaken to evaluate the effect of TRIP13 on the in vivo metastasis ability of gastric cancer cells in mice. Forty days after injection into the tail vein of mice, lungs were collected for H&E staining experiments. The results indicate that mice knocked down TRIP13 had fewer and smaller lung nodules. In contrast, the number and size of lung nodules in mice that rescued TRIP13 expression were significantly larger than in mice that knocked down TRIP13 (Fig. 3O, P), demonstrating that TRIP13 might promote metastasis of gastric cancer via triggering the EMT process.

### HDAC1 is a direct transcriptional activator of TRIP13

**Fig. 4:**
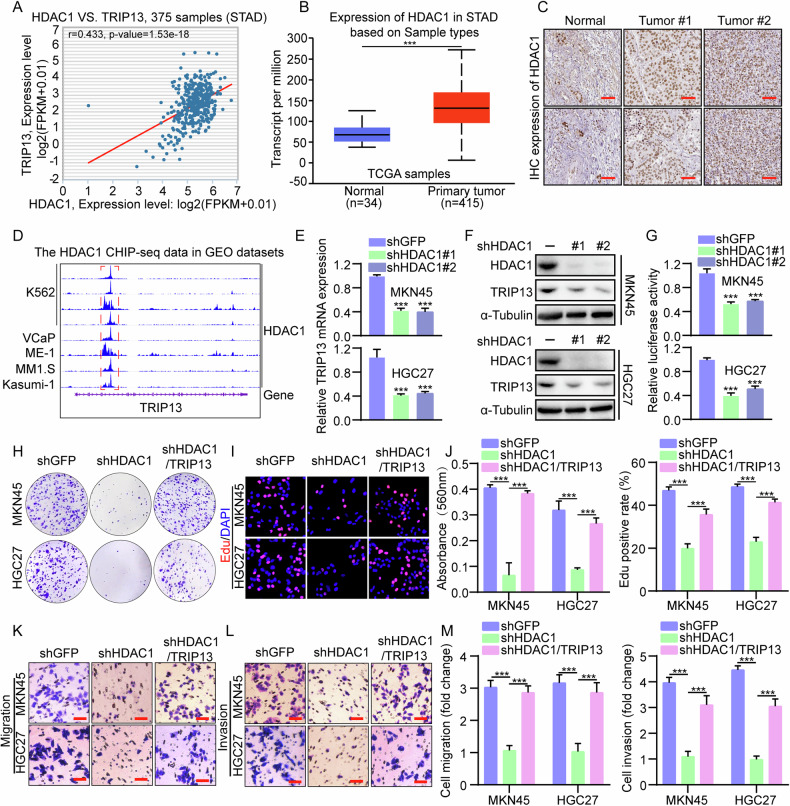
HDAC1 is a direct transcriptional activator of TRIP13. **A** The ENCORI database (https://rnasysu.com/encori/) was adopted to detect the correlation between HDAC1 and TRIP13 in gastric cancer. **B** UALCAN database (https://ualcan.path.uab.edu/cgi-bin/ualcan-res.pl) was performed to detect the expression of HDAC1 in normal gastric mucosal tissue and gastric cancer patient samples. **C** Protein Atlas database (http://www.proteinatlas.org/) was adopted to check expression of TTRIP13 in human non-tumor gastric mucosa and gastric cancer samples. **D** The Cistrome database (http://cistrome.org/db/#/) was used to analyze the enrichment of HDAC1 in the TRIP13 promoter region in different cells. **E**, **F** Fluorescence quantitative PCR and Western blot experiments were used to detect the expression of TRIP13 in MKN45 and HGC27 cells with HDAC1 knockdown. **G** The dual luciferase reporter gene experiment was conducted to detect the effect of knocking down HDAC1 on TRIP13 transcriptional activity. **H**–**J** The flat clone formation assay and Edu assay were used to detect the effect of overexpression of TRIP13 after knocking down HDAC1 on the hyperplasia ability of gastric cancer cells. **K**–**M** Transwell assay was performed to measure the influence of overexpression of TRIP13 after knocking down HDAC1 on migration and invasion ability of gastric cancer cells. All data were expressed as mean ± SD. The student’s *t*-test was performed to analyse the significance. *p < 0.05, **p < 0.01, ***p < 0.001.

To further investigate the mechanism of abnormal activation of TRIP13 in gastric cancer, a significant positive correlation between histone deacetylase 1 (HDAC1) and TRIP13 through analysis of the ENCORI database (Fig. 4A) was considered, inferring that HDAC1 might act as an upstream factor of TRIP13 to activate the expression of the latter. To verify the hypothesis, Chip-seq database analysis of the Cistrome DB database demonstrates that HDAC1 could bind to the promoter region of TRIP13 (Fig. 4D). Furthermore, analysis of the UALCAN database and the human protein atlas database uncovered that HDAC1 is highly expressed in the transcriptional level and patient tissue samples of gastric cancer (Fig. 4B, C). To further validate the regulation of TRIP13 by HDAC1, we constructed stable transgenic cell lines targeting knockdown of HDAC1. Western blot assay and fluorescence quantitative PCR assay indicated that knockdown of HDAC1 significantly inhibited the expression of TRIP13 (Fig. 4E, F). additionally, the dual luciferase reporter gene assay revealed that HDAC1 could regulate the transcriptional activity of TRIP13 (Fig. 4G). The effects of knocking down HDAC1 on the proliferation ability of gastric cancer cells were detected through plate clone formation and Edu experiments. The results indicated that knocking down the expression of HDAC1 significantly repressed the multiplication of gastric cancer cells (Fig. 4H–J). Transwell assays also revealed that knocking down HDAC1 significantly restrained the migration and invasion ability of gastric cancer cells (Fig. 4K–M). The flat panel clone formation assay, Edu assay, and transwell experiment revealed that knocking down the expression of HDAC1 and overexpressing TRIP13 could rescue the inhibition of proliferation, migration, and invasion ability of gastric cancer cells caused by knocking down HDAC1 (Fig. 4H–M). These experimental results confirm that HDAC1 binds to the promoter region of TRIP13 to regulate its transcriptional activity.

### TRIP13 interacts with DDX21 and correlates positively in tissue pathology

**Fig. 5:**
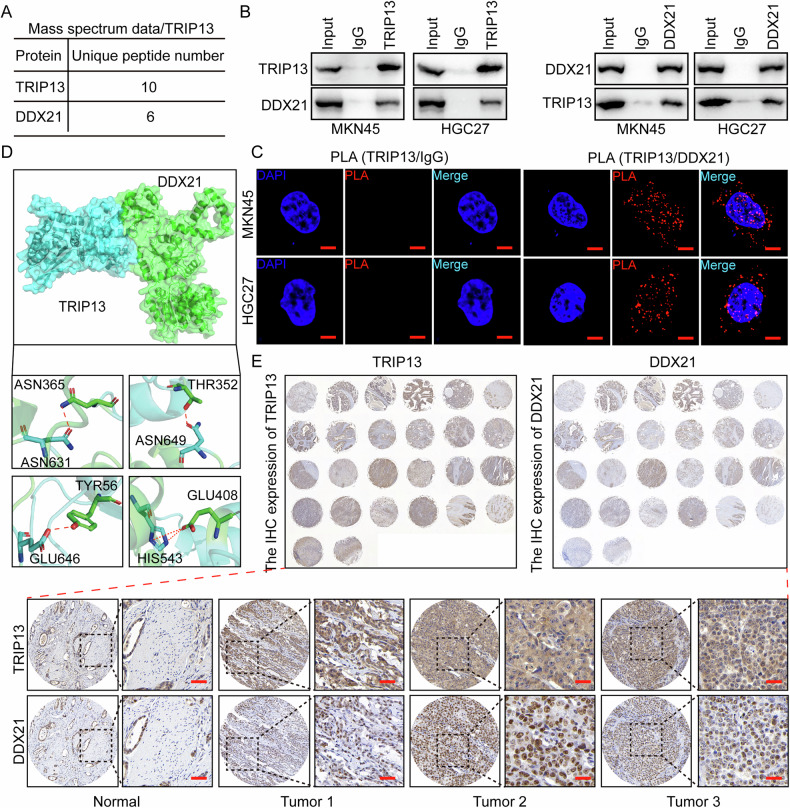
TRIP13 interacts with DDX21 and correlates positively in tissue pathology. **A** The protein mass spectrometry of TRIP13 and the number of DDX21 binding peptide segments. **B** Interaction between TRIP13 and DDX21 in MKN45 and HGC27 cells. **C** PLA assay was used to detect the interaction between TRIP13 and DDX21 in MKN45 and HGC27 cells. **D** Molecular dynamics analysis was performed to predict the amino acid sites where TRIP13 and DDX21 bind to each other. **E** Immunohistochemical experiments were performed to detect the correlation between TRIP13 and DDX21 in gastric cancer tissue samples.

Mass spectrometry analysis was undertaken further to investigate the molecular mechanisms of TRIP13 in gastric cancer. Mass spectrometry analysis revealed that DDX21 might serve as a binding protein for TRIP13 (Fig. 5A). Therefore, the interaction between TRIP13 and DDX21 was validated through immunoprecipitation assays. The experiment results confirmed the mutual binding of TRIP13 and DDX21 (Fig. 5B). Furthermore, proximity ligation assay (PLA) was performed to confirm further the binding between TRIP13 and DDX21 (Fig. 5C). Given the interaction between TRIP13 and DDX21, molecular dynamics simulation techniques were employed to analyse the specific amino acid sites at which bind (Fig. 5D). The above experiment confirms the mutual combination of TRIP13 and DDX21. An immunohistochemical assay was adopted to investigate the expression of TRIP13 and DDX21 in the same gastric cancer tissue samples. The experimental results identify a significant positive correlation between TRIP13 and DDX21 in gastric cancer tissue samples (Fig. 5E). These experimental results reveal that DDX21, as a binding protein of TRIP13, promotes the development of gastric cancer through its interaction with TRIP13.

### TRIP13 restrains the ubiquitination degradation of DDX21

**Fig. 6:**
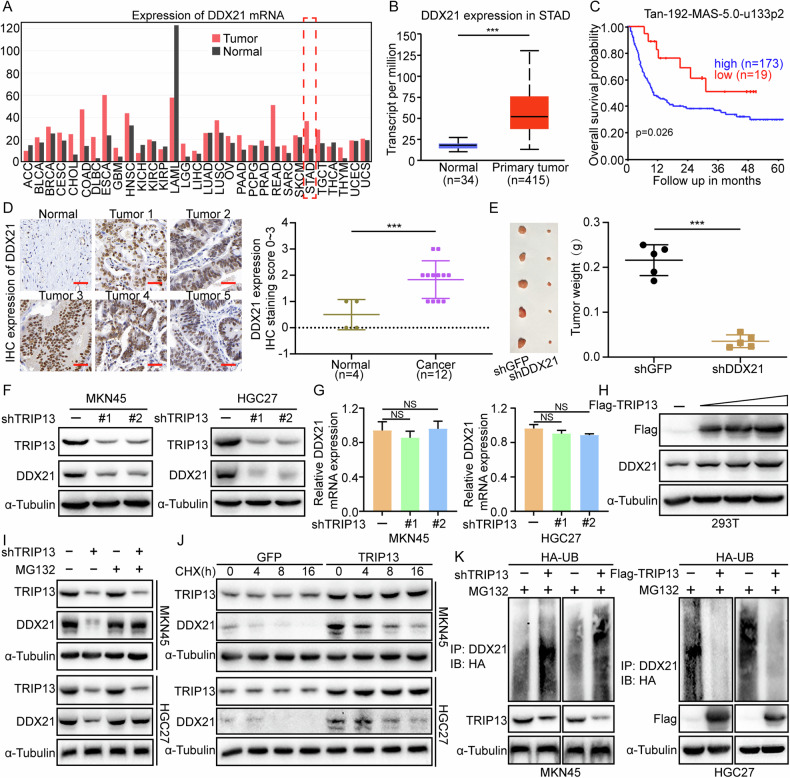
TRIP13 restrains the ubiquitination degradation of DDX21. **A** The GEPIA database (http://gepia.cancer-pku.cn/) was employed to check the expression of DDX21 in normal gastric mucosal tissue and gastric cancer samples. **B** UALCAN database (https://ualcan.path.uab.edu/cgi-bin/ualcan-res.pl) was performed to measure the expression of DDX21 in normal gastric mucosal tissue and gastric cancer patient samples. **C** The R2 database was applied to detect prognostic significance of DDX21. **D** Immunohistochemical experiment was performed to detect the expression of DDX21 in normal gastric mucosal tissue and gastric cancer tissue samples. **E** The mice subcutaneous tumorigenesis assay was used to detect the effect of knocking down the expression of DDX21 on the subcutaneous tumorigenesis ability of gastric cancer cells. **F**, **G** Western blot and Fluorescence quantitative PCR experiments were used to detect the expression of DDX21 in MKN45 and HGC27 cells with TRIP13 knockdown. **H** Western blot experiment was used to detect the effect of transient transfection of different doses of Flag-TRIP13 plasmid on DDX21 protein expression in 293T cells. **I** Cell lysates were prepared from knocked down TRIP13 cells that had been previously treated with or without MG132 for 8 h. **J** The DDX21 turnover rate of MKN45 and HGC27 cells overexpressing TRIP13. **K** Ubiquitination assay of DDX21 in both TRIP13-knocked-down MKN45 and HGC27 cells and MKN45 and HGC27 cells overexpressing TRIP13. Equal amounts of cell lysates were immunoblotted with the indicated antibodies. All data were expressed as mean ± SD. The student’s *t*-test was performed to analyse the significance. *p < 0.05, **p < 0.01, ***p < 0.001.

Studies have shown that DDX21 is abnormally expressed in gastric cancer. Investigation of Gene Expression Profiling Interactive Analysis (GEPIA) and TCGA databases revealed that DDX21 is highly expressed in gastric cancer patients with poor prognoses (Fig. 6A, B). The analysis of the R2 prognostic database further confirms that patients with high expression of DDX21 in gastric cancer with poor prognosis (Fig. 6C). To further investigate the expression of DDX21 in gastric cancer, immunohistochemical assay was conducted to detect the expression of DDX21 in normal gastric mucosal tissue and gastric cancer tissue samples. The results of the immunohistochemistry assay demonstrate that DDX21 is highly expressed in gastric cancer tissue samples (Fig. 6D). Additionally, small interfering RNA was designed to knock down the expression of DDX21 to detect its effect on the subcutaneous tumourigenesis ability of gastric cancer cells in mice. The experimental results confirm that knocking down the expression of DDX21 could significantly restrain the subcutaneous tumourigenesis ability of gastric cancer cells in mice (Fig. 6E). We previously demonstrated that TRIP13 interacts with DDX21. To further explore the regulatory effect of TRIP13 on DDX21, we assessed, via western blot and qRT-PCR assays, the relative expression level of DDX21. We found that DDX21 protein expression was reduced in TRIP13 knocked-down gastric cancer cells (Fig. 6F). However, the mRNA expression of DDX21 was not significantly changed in TRIP13 knocked-down cells (Fig. 6G). Hence, we assumed that TRIP13 could regulate the expression of DDX21 via ubiquitination. The observation results indicated that the expression of DDX21 increased in a dose-dependent manner with TRIP13 (Fig. 6H). To further detect whether TRIP13 regulates DDX21 ubiquitination, knocked-down MKN45, and HGC27 cells were treated with MG132. The findings obtained demonstrate rescue of DDX21 protein expression level (Fig. 6I). Moreover, the use of de novo protein synthesis inhibitor cycloheximide (CHX) to detect the DDX21 turnover rate demonstrated that the DDX21 degradation rate was slowed down in gastric cancer cells overexpressing TRIP13 (Fig. 6J). The ubiquitination assay was performed to confirm that knocking down TRIP13 increased the ubiquitination degradation of DDX21, while overexpression of TRIP13 repressed the ubiquitination degradation of DDX21 (Fig. 6K). Substantially, these results reveal that DDX21 plays the oncogene role in gastric cancer and TRIP13 could restrain the ubiquitination degradation of DDX21.

### TRIP13 promotes the progression of gastric cancer through DDX21

**Fig. 7:**
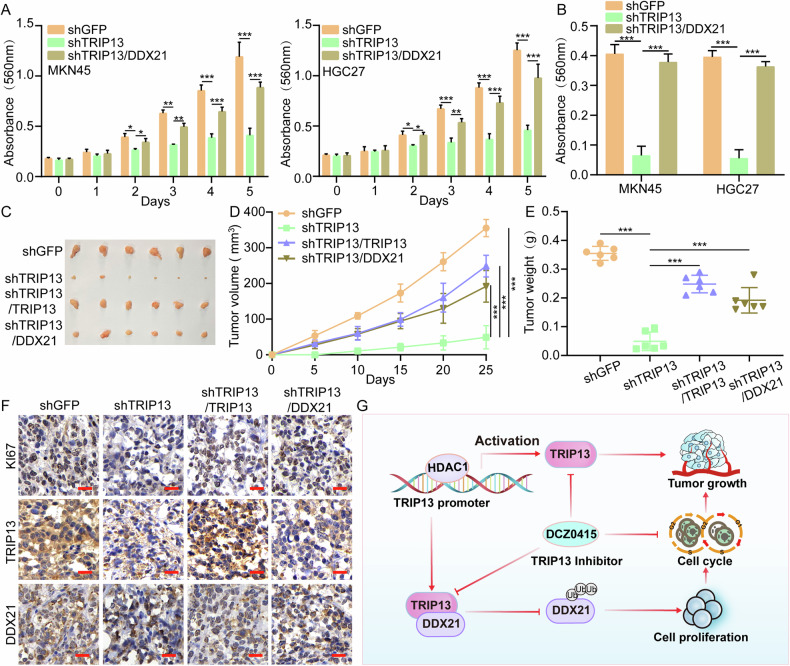
TRIP13 promotes the progression of gastric cancer through DDX21. **A** The MTT assay was used to detect the impact of knocking down TRIP13 and overexpressing DDX21 on the multiplication ability of gastric cancer cells. **B** The plate colony formation ability after overexpression of DDX21 in TRIP13-knocked-down MKN45 and HGC27 cells. **C**–**E** The volume and size of subcutaneous tumors in mice after overexpression of TRIP13 and DDX21 in TRIP13-knocked-down MKN45 cells. **F** Expression levels of Ki67, TRIP13 and DDX21 were detected by immunohistochemistry. **G** HDAC1 mediates TRIP13/DDX21 axis to promote the development of gastric cancer. All data were expressed as mean ± SD. The student’s *t*-test was performed to analyse the significance. *p < 0.05, **p < 0.01, ***p < 0.001.

To investigate the effects of knocking down TRIP13 and overexpressing DDX21 on the proliferation ability of gastric cancer cells. The MTT and flat clone formation assays were adopted to detect the effect of overexpression of DDX21 after knocking down TRIP13 on the proliferation ability of gastric cancer cells. The results revealed that overexpression of DDX21 after knocking down TRIP13 rescued the proliferation restrain of gastric cancer cells caused by knocking down TRIP13 (Fig. 7A, B). Furthermore, the subcutaneous tumour formation experiment demonstrated that knocking down TRIP13 significantly reduced the volume, weight, and growth curve of subcutaneous tumours in mice. After rescuing TRIP13, the size and weight of subcutaneous tumours in mice partially recovered (Fig. 7C–E). Finally, the subcutaneous tumour formation experiment in mice confirmed that overexpression of DDX21 after knocking down TRIP13 could partially restore the inhibition of subcutaneous tumours in mice caused by knocking down TRIP13 (Fig. 7C–E). Immunohistochemical experiments also showed that the positive rates of TRIP13, DDX21, and Ki67 were significantly reduced in the TRIP13 knockdown tumour group, while after rescuing the expression of TRIP13, the positive rates of TRIP13, DDX21, and Ki67 were significantly restored (Fig. 7F). Additionally, in the tumour group overexpressing DDX21 after knocking down TRIP13, the positive rates of DDX21 and Ki67 significantly recovered (Fig. 7F). The proposed model and mechanistic diagram through which TTRIP13 regulates DDX21 (Fig. 7G).

## Discussion

GC (Gastric cancer) is one of the most common cancers and the third leading cause of cancer deaths worldwide. Over one million patients worldwide are diagnosed with gastric cancer each year [2, 36]. At present, the main treatment methods for gastric cancer patients are surgery and systemic chemotherapy, and radiation therapy, immunotherapy, and targeted therapy are gradually being applied [37]. However, the 5-year survival rate of gastric cancer patients is still not ideal. Therefore, early detection of GC plays a crucial role in the treatment and prognosis of GC [38]. Better elucidating the pathogenesis of cancer, searching for biomarkers for early cancer screening, and exploring new targets for cancer treatment are urgent issues that need to be addressed in current cancer treatment. Our research results indicated that TRIP13 is in an abnormally activated state in gastric cancer, and patients with high expression of TRIP13 have poor prognosis, revealing that TRIP13 might play an essential role in the tumour development process of gastric cancer. To investigate the mechanism of TRIP13 in gastric cancer, TRIP13 was knocked down using a shRNA sequence, illustrating that downregulating TRIP13 significantly inhibits gastric cancer cell proliferation, migration, invasion, tumourigenesis and metastasis.

TRIP13 was initially discovered to be one of several proteins interacting with thyroid hormone receptors, which belongs to the protein family AAA+ ATPase. The AAA+ ATPase family members perform various cellular functions, including proteasome action, membrane transport, DNA replication, and molecular motor movement [39–41]. Studies have shown that TRIP13 plays a vital role in mitosis. The maintenance of genomic stability during mitosis is coordinated by the spindle assembly checkpoint (SAC) through its effector mitotic checkpoint complex (MCC), which is an inhibitor of the anaphase-promoting complex (APC/C, also known as the cyclosome) [42, 43]. The MCC comprises BUBR1, BUB3, CDC20, and closed MAD2 (C-MAD2) [44–48]. The critical component controlling MCC assembly is MAD2, an essential checkpoint protein containing a HORMA domain [49] that could adopt two different conformations: “open” (O-Mad2) or “closed” (C-Mad2). Research reports reveal that TRIP13 synergises with p31 to promote the release of MAD2 in the (mitotic checkpoint complex) MCC, thereby relieving the checkpoint inhibition of the ubiquitin ligase (Anaphase-Promoting Complex/Cyclosome) APC/C, which mediates ubiquitination modification of cell cycle-related proteins [50] and inactivates the mitotic checkpoint [51, 52]. Notably, studies have shown that the conformation of MAD2 in vivo is highly dependent on TRIP13 and its ATPase activity. After the loss of TRIP13, insufficient soluble O-MAD2 during mitosis could lead to failure of the mitotic checkpoint activation, and checkpoint activation can be rescued by reintroducing additional O-MAD2 [53]. The above research results confirm the dual role of TRIP13 in activating and silencing mitotic checkpoints. The dual role of TRIP13 in mitotic checkpoints reflects the similar duality observed in cancer. The current study confirms that TRIP13 is highly expressed in gastric cancer and participates in the proliferation, tumourigenesis and metastasis of gastric cancer cells. Mechanistically, HDAC1 is an upstream factor of TRIP13, and a double luciferase assay was performed to confirm that HDAC1 regulates the transcriptional activity of TRIP13. Further research revealed that TRIP13 interacted with DDX21 and is significantly positively correlated in gastric cancer tissue samples. Finally, TRIP13 was found to restrain the ubiquitination degradation of DDX21, promoting the progression of gastric cancer. It is precisely due to the dual role of TRIP13 at the mitotic checkpoint complex (MCC) that the hypothesis of whether the absence of TRIP13 might mediate the silencing of the mitotic checkpoint complex (MCC) and activate APC/C-mediated degradation of cycle-related proteins to restrain gastric cancer cell proliferation arose. As a member of the AAA+ ATPase family, TRIP13 could mediate the modification of proteasomes. However, TRIP13 is not a ubiquitination ligase, so potentially, the inhibition of DDX21 ubiquitination degradation mediated by TRIP13 requires the involvement of ubiquitination ligases, which requires further investigation.

Overall, this study confirms that TRIP13 is involved in the proliferation, migration, invasion in vitro and tumourigenesis and metastasis in vivo of gastric cancer cells. Mechanistically, HDAC1 activates the transcription of TRIP13 as an upstream factor, and the interaction between TRIP13 and DDX21 represses the ubiquitination degradation of the latter. These results suggest that the HDAC1-TRIP13/DDX21 axis might provide a solid theoretical basis for the clinical treatment of gastric cancer patients, and TRIP13 might serve as a therapeutic target for gastric cancer patients.

### Supplementary information

Supplementary Figures and Tables

Full and uncropped western blots

## Data Availability

This article and the Supplementary Information file include all data analysed or generated in this study.
