# Supplementary material for: TRIP13 regulates progression of gastric cancer through stabilising the expression of DDX21
Source: Cell Death Dis. 2024 Aug 26;15(8):622. doi: 10.1038/s41419-024-07012-x (PMC11347623; doi:10.1038/s41419-024-07012-x)
Supplement: Supplementary file 1 — Supplementary Figures and Tables [file 41419_2024_7012_MOESM1_ESM.docx]

**
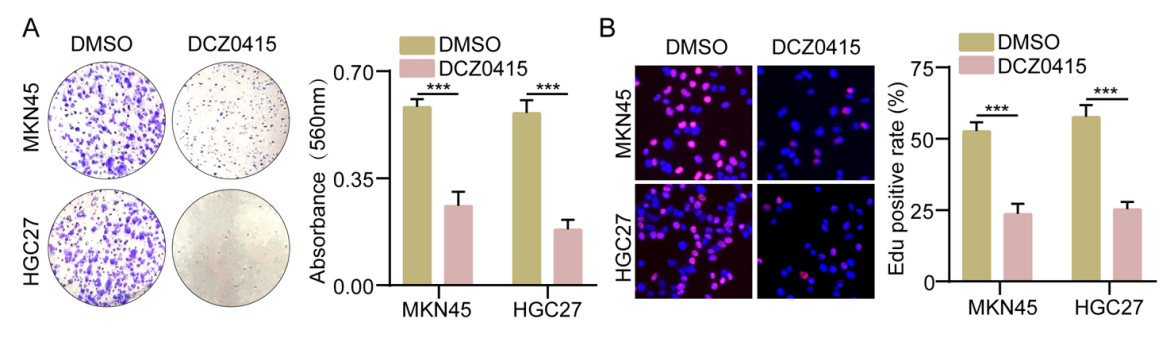
Supplementary Figures**


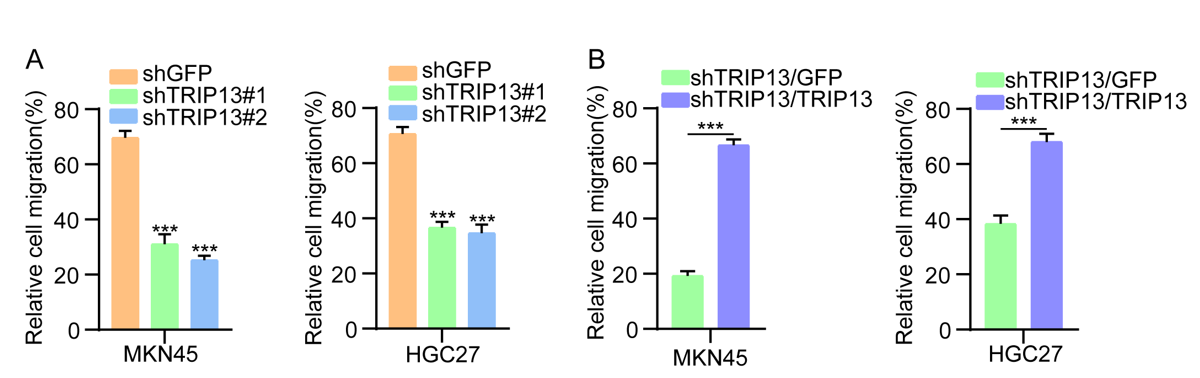
**Supplementary Figure 1: Small molecule inhibitor DCZ0415 restrains the proliferation of gastric cancer cells. A, B** The plate cloning formation experiment and Edu experiment were conducted to detect the effect of DCZ0415 on the proliferation ability of gastric cancer cells. All data were expressed as mean ± SD. The student’s t test was performed to analyse the significance. *p < 0.05, **p < 0.01, ***p < 0.001.

**Supplementary Figure 2: TRIP13 facilitates gastric cancer cell migration ability. A** Wound-healing assay was performed with TRIP13-knockdown MKN45 and HGC27 cells. **B** Wound-healing experiment was conducted in gastric cancer cells that rescued TRIP13 expression after knocking down TRIP13. All data were expressed as mean ± SD. The student’s t test was performed to analyse the significance. *p < 0.05, **p < 0.01, ***p < 0.001.


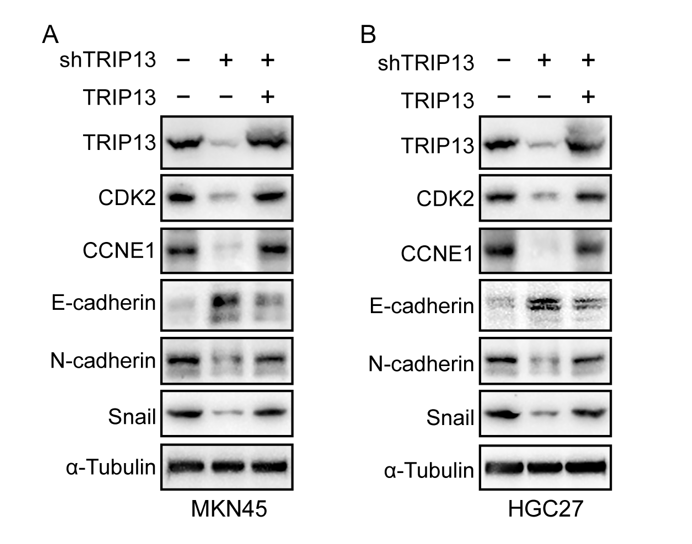


**Supplementary Figure 3: TRIP13 activates gastric cancer cell-cycle related proteins, migration and invasion related proteins. A, B** Western blot experiments were performed to detect the protein expression of TRIP13, CDK2, CCNE1, E-cadherin, N-cadherin and Snail in gastric cancer cells that were knocked down and rescued after knocking down TRIP13.

**RT-PCR primers Table 1**

| TRIP13 Forward (5′−3′) | TGTGTAAAGCGTTAGCCCAGA |
| --- | --- |
| TRIP13 Reverse (5′−3′) | GCCACTTTCCGAAAACCACTTA |
| DDX21 Forward (5′−3′) | TCATCAAGGACGCACTATCATCT |
| DDX21 Forward (5′−3′) | CCTTTCAGGGTGATTTCCCTTT |

**shRNA primers Table 2**

| shTRIP13 Forward#1 (5′−3′) | CCGGGCTACTCAACAGACATAATATCTCGAGAT  ATTATGTCTGTTGAGTAGCTTTTTG |
| --- | --- |
| shTRIP13 Reverse#1 (5′−3′) | AATTCAAAAAGCTACTCAACAGACATAATATCTC  GAGATATTATGTCTGTTGAGTAGC |
| shTRIP13 Forward#2 (5′−3′) | CCGGATGCTGTCTTGACCCAAATTGCTCGAGCA  ATTTGGGTCAAGACAGCATTTTTTG |
| shTRIP13 Reverse#2 (5′−3′) | AATTCAAAAAATGCTGTCTTGACCCAAATTGCTC  GAGCAATTTGGGTCAAGACAGCAT |
| shHDAC1 Forward#1 (5′−3′) | CCGGGCCGGTCATGTCCAAAGTAATCTCGAGAT  TACTTTGGACATGACCGGCTTTTTG |
| shHDAC1 Reverse#1 (5′−3′) | AATTCAAAAAGCCGGTCATGTCCAAAGTAATCTC  GAGATTACTTTGGACATGACCGGC |
| shHDAC1 Forward#2 (5′−3′) | CCGGCCACAGCGATGACTACATTAACTCGAGTTA  ATGTAGTCATCGCTGTGGTTTTTG |
| shHDAC1 Reverse#2 (5′−3′) | AATTCAAAAACCACAGCGATGACTACATTAACTCG  AGTTAATGTAGTCATCGCTGTGG |
| shDDX21 Forward (5′−3′) | CCGGCGCTCCTTGATCAACTCAAATCTCGAGATT  TGAGTTGATCAAGGAGCGTTTTTG |
| shDDX21 Reverse (5′−3′) | AATTCAAAAACGCTCCTTGATCAACTCAAATCTCG  AGATTTGAGTTGATCAAGGAGCG |
