# Supplementary figures and images for: TRIP13 regulates progression of gastric cancer through stabilising the expression of DDX21
Source: Cell Death Dis. 2024 Aug 26;15(8):622. doi: 10.1038/s41419-024-07012-x (PMC11347623; doi:10.1038/s41419-024-07012-x)

Figure 2

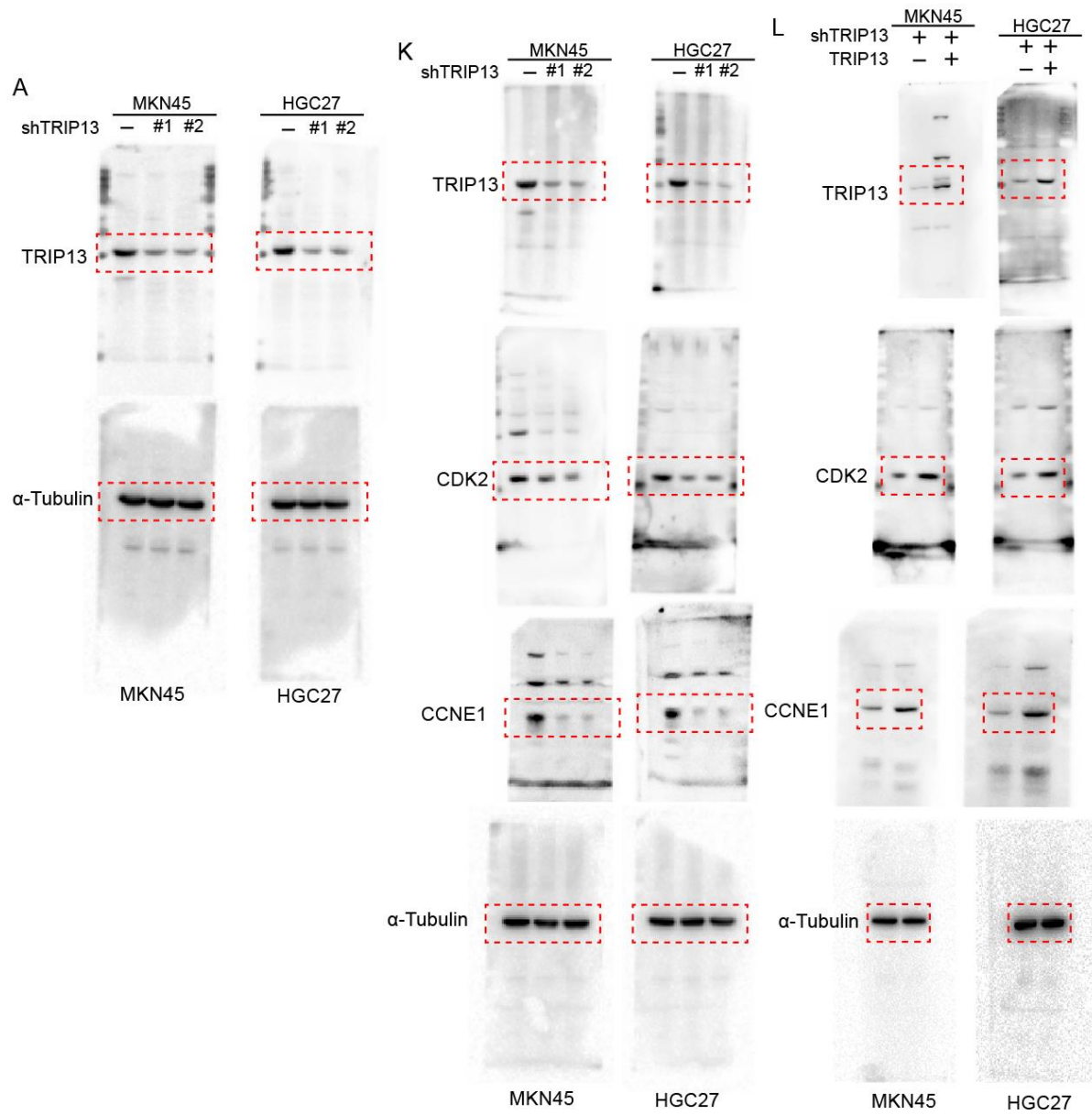

Figure 3

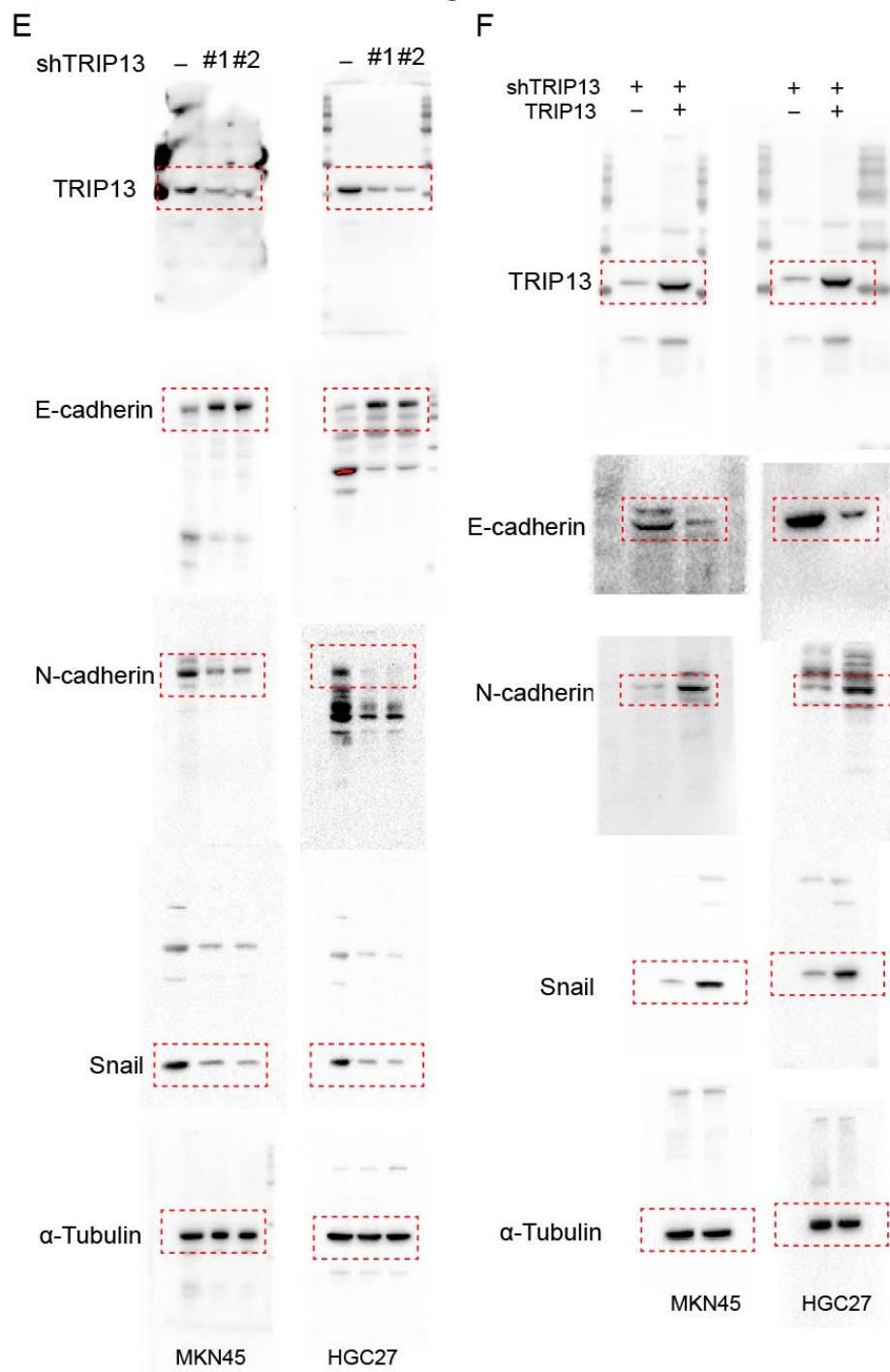

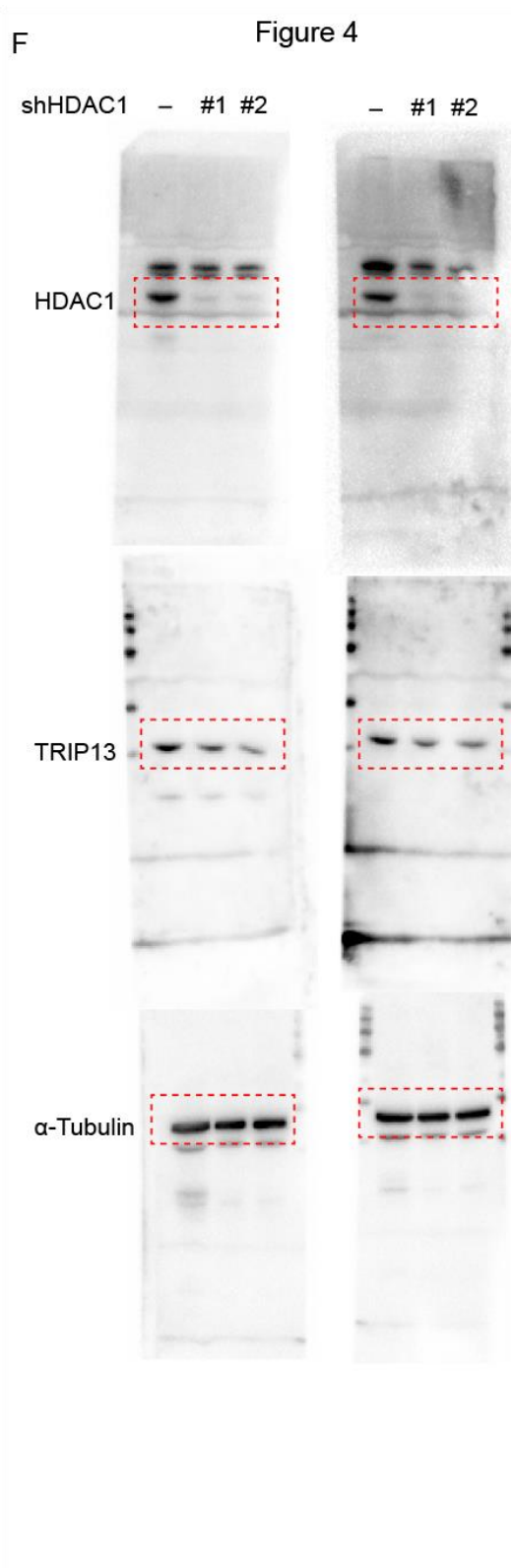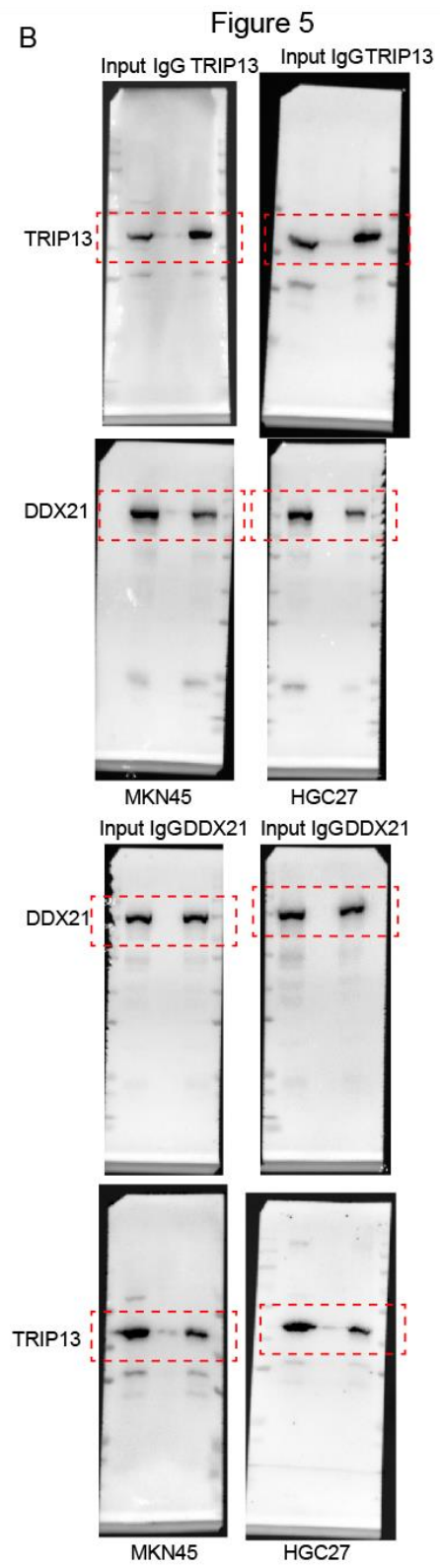

Figure 6

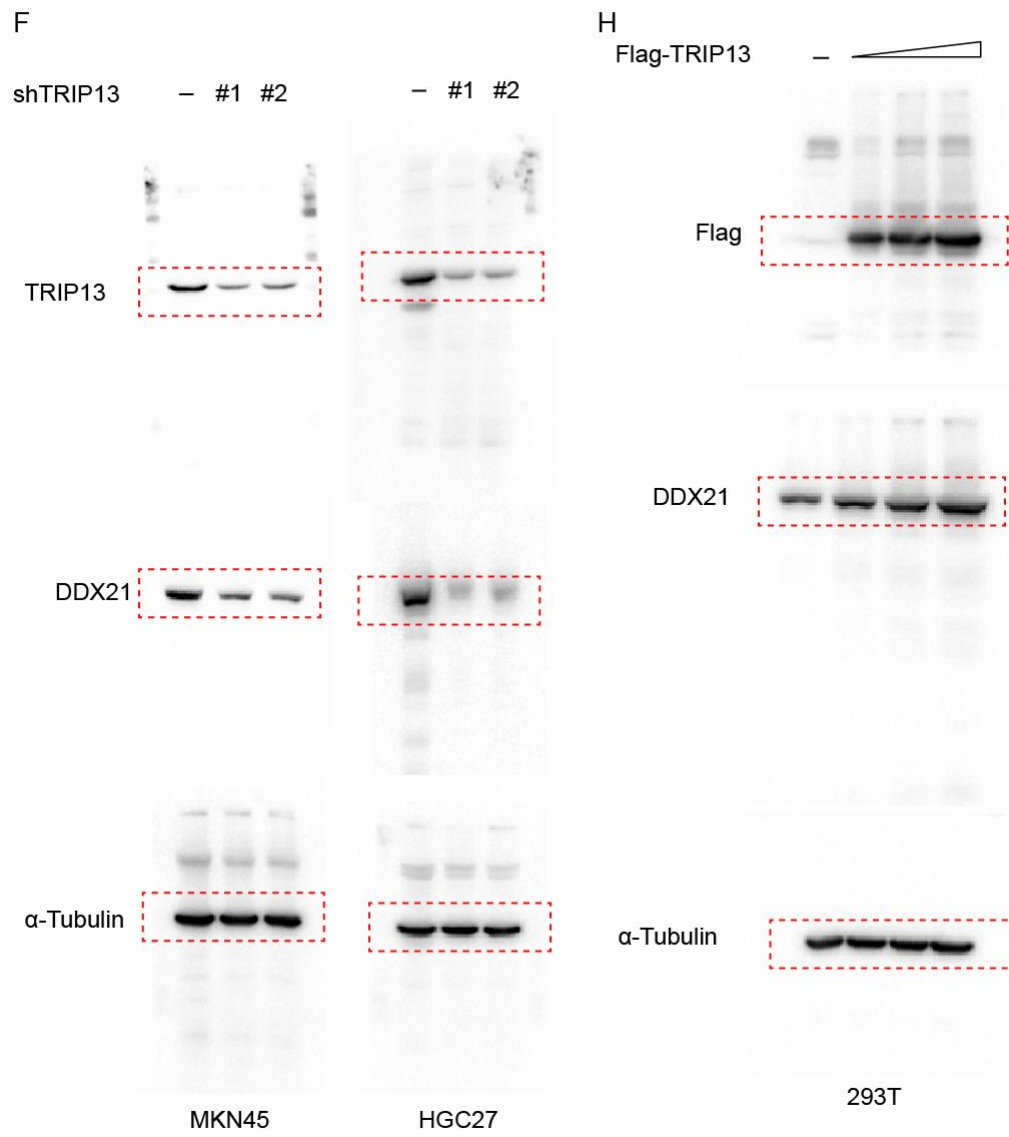

Figure 6

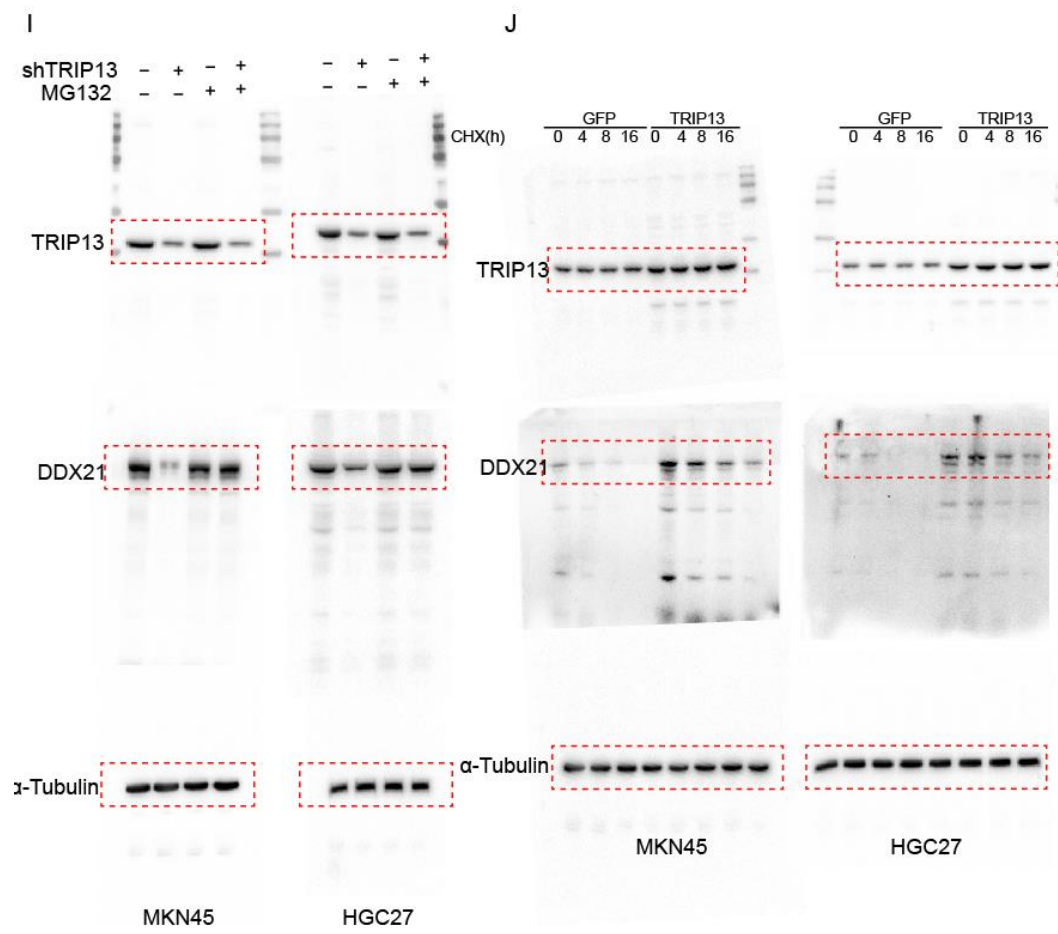

Figure 6

K

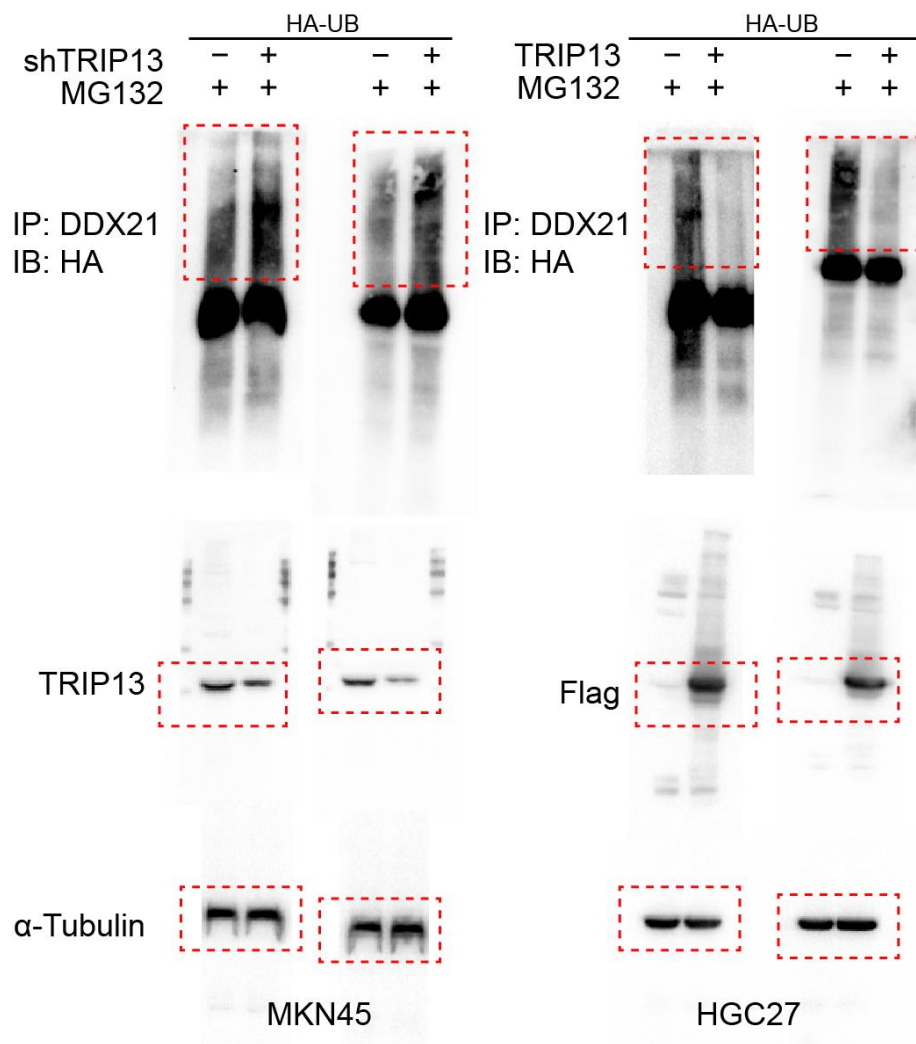

Figure S 3A, B

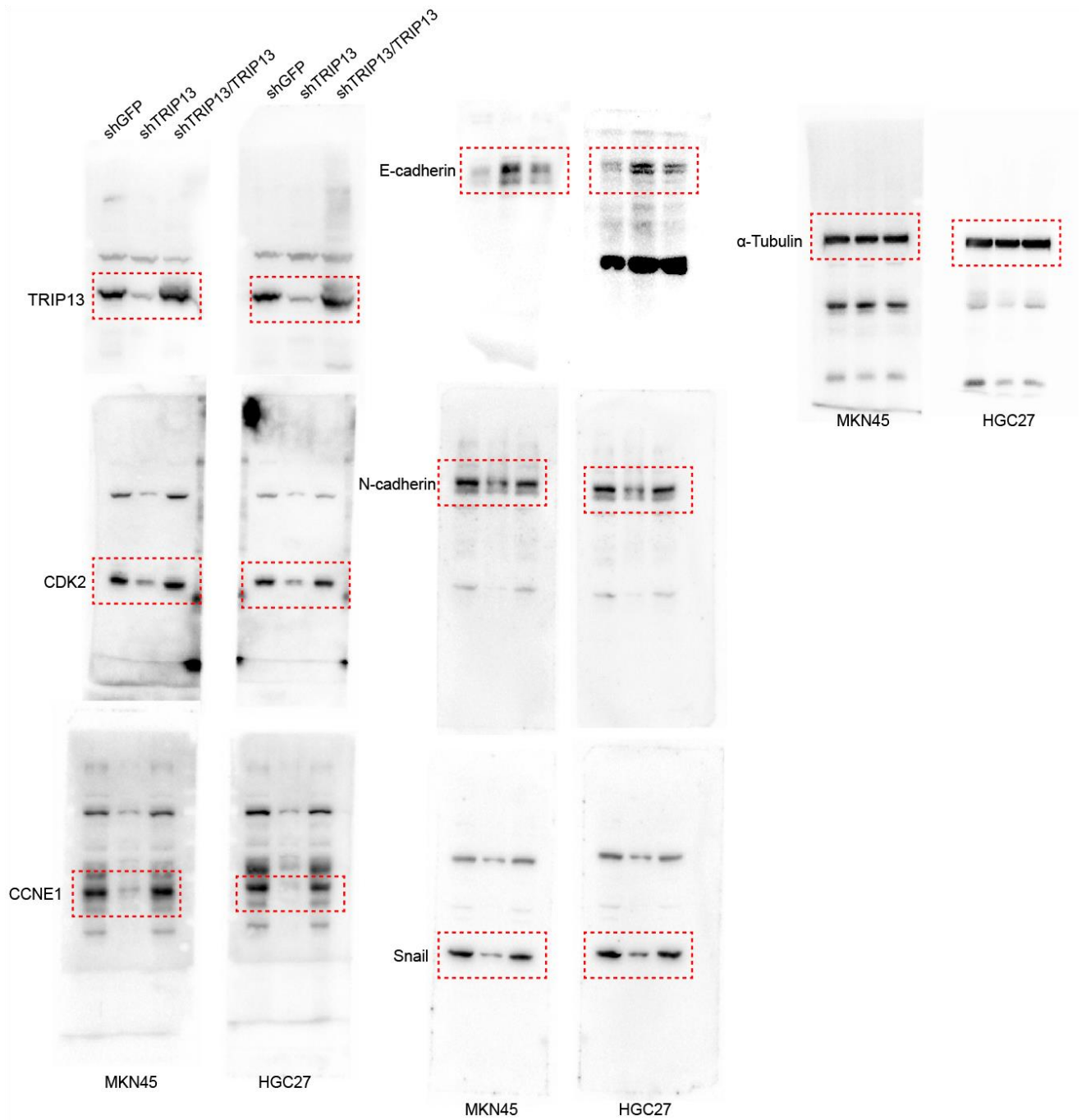

Supplement: Supplementary file 2 — Full and uncropped western blots [file 41419_2024_7012_MOESM2_ESM.pdf]
